# Doublecortin-expressing cell types in temporal lobe epilepsy

**DOI:** 10.1186/s40478-018-0566-5

**Published:** 2018-07-13

**Authors:** Joan Y. W. Liu, Mar Matarin, Cheryl Reeves, Andrew W. McEvoy, Anna Miserocchi, Pamela Thompson, Sanjay M. Sisodiya, Maria Thom

**Affiliations:** 1 Department of Clinical and Experimental Epilepsy, UCL Institute of Neurology, Queen Square, London, WCN1BG UK; 2 Department of Neuropathology, National Hospital for Neurology and Neurosurgery, UCL Institute of Neurology, Queen Square, London, WC1N 3BG UK; 3 Department of Neurology, National Hospital for Neurology and Neurosurgery, London, UK; 4 Department of Neuropsychology, National Hospital for Neurology and Neurosurgery, London, UK; 5 Department of Neurosurgery, National Hospital for Neurology and Neurosurgery, London, UK; 6 Chalfont Centre for Epilepsy, Chesham Lane, Chalfont St Peter, Buckinghamshire, SL9 0RJ UK; 7 Department of Biomedical Sciences, University of Westminster, W1W6UW, London, UK

**Keywords:** Doublecortin, Temporal lobe epilepsy, Hippocampus, Memory, Microglia

## Abstract

Doublecortin (DCX) is widely regarded as a marker of immature and migrating neurons during development. While DCX expression persists in adults, particularly in the temporal lobe and neurogenic regions, it is unknown how seizures influence its expression. The aim of the present study was to explore the distribution and characteristics of DCX-expressing cells in surgical and postmortem samples from 40 adult and paediatric patients, with epilepsy and with or without hippocampal sclerosis (HS), compared to post mortem controls. The hippocampus (pes and body), parahippocampal gyrus, amygdala, temporal pole and temporal cortex were examined with DCX immunohistochemistry using four commercially-available DCX antibodies, labelled cells were quantified in different regions of interest as well as their co-expression with cell type specific markers (CD68, Iba1, GFAP, GFAP∂, nestin, SOX2, CD34, OLIG2, PDGFRβ, NeuN) and cell cycle marker (MCM2). Histological findings were compared with clinical data, as well as gene expression data obtained from the temporal cortex of 83 temporal lobe epilepsy cases with HS. DCX immunohistochemistry identified immature (Nestin^−^/NeuN^−^) neurons in layer II of the temporal neocortex in patients with and without epilepsy. Their number declined significantly with age but was not associated with the presence of hippocampal sclerosis, seizure semiology or memory dysfunction. DCX^+^ cells were prominent in the paralaminar nuclei and periamygdalar cortex and these declined with age but were not significantly associated with epilepsy history. DCX expressing cells with ramified processes were prominent in all regions, particularly in the hippocampal subgranular zone, where significantly increased numbers were observed in epilepsy samples compared to controls. DCX ramified cells co-expressed Iba1, CD68 and PDGFRβ, and less frequently MCM2, OLIG2 and SOX2, but no co-localization was observed with CD34, nestin or GFAP/GFAP ∂. Gene expression data from neocortical samples in patients with TLE and HS supported ongoing DCX expression in adults. We conclude that DCX identifies a range of morphological cell types in temporal lobe epilepsy, including immature populations, glial and microglial cell types. Their clinical relevance and biological function requires further study but we show some evidence for alteration with age and in epilepsy.

**Electronic supplementary material:**

The online version of this article (10.1186/s40478-018-0566-5) contains supplementary material, which is available to authorized users.

## Introduction

Doublecortin (DCX) is a microtubule-associated protein critical for normal neuronal migration during development. It has been widely used as a reliable marker to study post-mitotic, immature neurons in the adult mammalian brain [11, 14, 33, 37] as well as responses of these cell types to brain insults [9, 25, 51]. There is anatomically restricted expression in the normal mature mammalian brain, with DCX^+^ multipolar and ‘tangled’ neurons reported in cortical layer II, mainly in the temporal lobe, in a variety of species [36, 49] and in the peri-amydgala association cortex and amygdala [52]. The physiological function of persisting DCX^+^ neurons is unknown: roles in olfactory processing and memory have been postulated [6]. DCX^+^ neurons remain relatively unexplored in humans and their clinical significance is uncertain. DCX^+^ populations diminish with age in animals [6] but studies have suggested seizure-enhanced maturation and proliferation of DCX^+^ cell types occurs in temporal lobe epilepsy (TLE) [9, 28, 40] indicative of their underlying plasticity and responsiveness. In addition, reports have noted DCX expression in non-neuronal cell types, including in relation to cortical pathology and brain injury and repair [25, 46].

The aim of this study was to further explore the morphology, phenotype, distribution and density of DCX^+^ cells in TLE. We included surgical samples from a wide age range both with and without hippocampal sclerosis (HS), the commonest pathology in TLE [4]. We compared this to findings in post-mortem (PM) samples from patients with epilepsy and HS and non-epilepsy controls to explore any differences in the morphology and number of DCX ^+^ cells between these groups. In addition we compared the neuropathological findings to available gene-expression data from a parallel study of the temporal lobe cortex from a large series of TLE patients. Neuropathology findings were also correlated with clinical epilepsy history, including type of seizures, aura and any memory dysfunction, to explore pathophysiological roles of these cell types.

## Methods

### Case selection

Fifty-two cases were included in the histological study (Table 1). Adult temporal lobe epilepsy (TLE) cases from patients undergoing elective surgery for the treatment of refractory epilepsy (*n* = 19) were selected from the databases of the Epilepsy Society Brain and Tissue Bank at UCL Institute of Neurology and pediatric TLE surgical cases (*n* = 5) were obtained from Great Ormond Street Hospital NHS Trust. Adult post mortem (PM) tissue from patients with (*n* = 16) or without (*n* = 12) epilepsy during life (healthy controls) was obtained from the Epilepsy Society and MRC sudden death brain banks respectively; none of these patients had undergone neurosurgical treatments during life. The study has ethical approval and all cases were consented for use in research (Ethics committee approval NRES17/SC/0573). In 18/24 surgical cases and 8/16 of the epilepsy PM cases, hippocampal sclerosis (HS) ILAE type 1 [5] was present. None of these cases had additional temporal lobe sclerosis/cortical dysplasia [43] or other lesion.

**Table 1:** Clinical and pathology details of cases and control groups (Further detail of each case is available in Additional file 1: Table S1)

Group	Tissue type	NUMBER	Predominant pattern of HS in body	Age (at surgery or death); mean (range, years)	Gender	Regions examined/study
Adult epilepsy TLE/HS (S1–13)	Surgical Fixed	13	ILAE Type 1 HS	41.3 (22–54)	7F: 6 M	HB,PES,PHG,TPole, TLobe, Amyg / qIHC
Paediatric epilepsy TLE/HS (S1–5)	Surgical Fixed	5	ILAE type 1 HS only	12.2 (8–15)	2F: 3 M	HB, TLobe / q IHC
Adult epilepsy TLE/NO HS (S19–24)	Surgical Fixed	6	NO HS	28.6 (24–35)	2F: 4 M	HB,PES,PHG,TPole, TLobe, Amyg / q IHC
Adult epilepsy (EPM1–16)	Post Mortem	16	ILAE type 1 HS in 8 CASES	48.7 (18–75)	8F: 8 M	HB, PHG, Amyg, Temporal Cortex (Both hemispheres included in 4 cases) / q IHC
Adult non-epilepsy controls (C1–12)	Post mortem	12	No HS	56.4 (28–85)	5F: 7 M	HB, PHG, Amyg, Temporal Cortex / q IHC
Adult epilepsy TLE/HS	Fresh	83 77^a^	All HS (16 with TLS) All HS (15 with TLS)	36.3 (16–63) 35.6 (16–57)	45F-38M 39F-38M	Middle temporal gyrus (cortex) / RNA
Adult non-epilepsy controls	Fresh	73^b^ 59^a^	No HS No HS	50.8 (20–79) 28.8 (13–55)	14F–53F 30F-29M	Middle temporal gyrus (cortex)/ RNA A1C, inferior and superior TC

*S* surgical epilepsy case, *EPM* adult epilepsy post-mortem, *C* Post-mortem control, *HB* Hippocampal body, *PES* pes hippocampus, *PHG* parahippocampal gyrus, *TPole* temporal pole, *TLobe* temporal lobe, *TLS* temporal lobe sclerosis, *Amyg* amygdala, *HS* hippocampal sclerosis, *qIHC* qualitative and quantitative immunohistochemistry, *RNAseq* RNA sequencing and expression analysis, *ILAE* International League against epilepsy, *TLE* temporal lobe epilepsy.

^a^Only samples from adolescence and young and middle adulthood period. Controls from Kang et al.

^b^Samples obtained from the MRC brain bank, Edinburgh. A1C = Primary auditory cortex

**Fig. 1:**
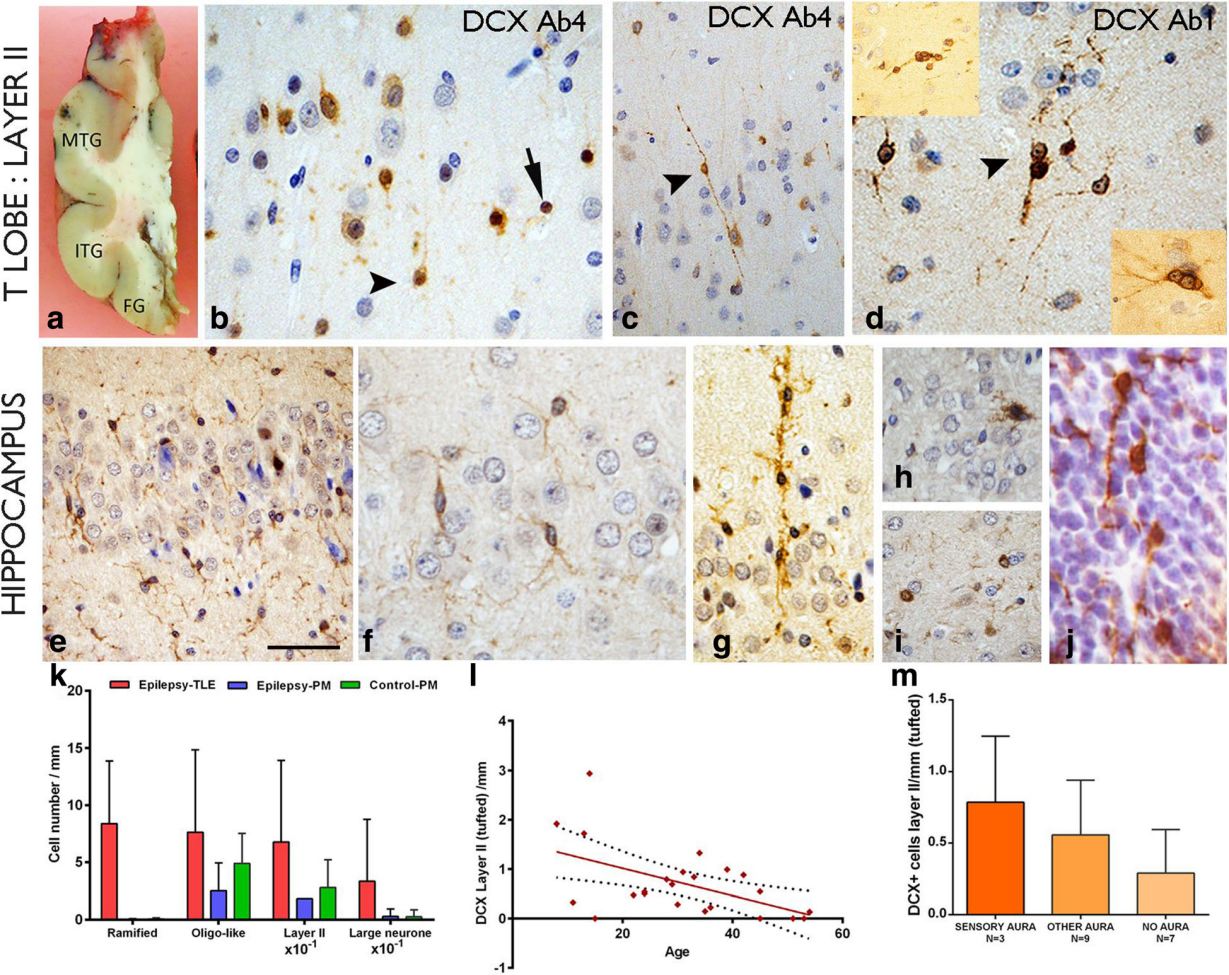
Doublecortin (DCX) in the cortex and hippocampus. **a** Section though a temporal lobe indicating the regions studies (MTG = middle temporal gyrus, ITG, inferior temporal gyrus, FG = fusiform gyrus) **b** Layer II DCX positive cells (DCX^+^) using DCX Ab 4 (see Table 2). Cells of different size, including some with more neuronal features and radial perpendicular processes (arrowhead) as well as dense nuclear labelling of small cells without processes (arrow) were observed. **C**. A bipolar cell in cortical layer II with DCX labelling with long beaded processes extending perpendicularly into layer I. **d** Clusters of small, intensely labelled DCX+ cells at interface of layer II and I labelled using DCX Ab1 (see Table 2). Top insert shows clusters of DCX^+^ cells; the bottom insert shows prominent nucleoli and neuronal appearance of DCX^+^ cells. **e** In the hippocampus granule cell layer (GCL) small DCX^+^ cells with ramified, multiple processes were observed; **f** In another case, the delicate branching processes of the ramified cells are shown. **g** A column of DCX^+^ cells extending though the GCL was observed in another case. **h** Granule cell neurons showed occasional DCX expression. **i** Small round DCX^+^ oligo-like cells were noted in the hippocampus in satellite location to neurons. **j**.DCX expression, in the periventricular germinal matrix of the lateral ventricle, in a developmental human control of 13 weeks, showing small cells with extended processes. **k** Bar chart showing greater linear densities for all morphological DCX^+^ cell types in surgical epilepsy cases compared to post mortem (PM) epilepsy controls and controls with statistically significant differences noted for ramified cell types only (*p* < 0.0001). **l** The linear density of layer II tufted DCX^+^ cell types showed an inverse correlation with age for all cases (surgical and PM) (*p* = 0.001) as well as for surgical cases alone (*p* = 0.016; not shown on graph). **m** Although greater DCX^+^ linear densities of tufted cells were present in patients with sensory aura of abnormal taste and smell compared to other aura types or no auras but these differences were not significant. Bar = 1 cm in A; B, D, F, G-J = 20 μm approx. (original magnification × 400) and C and E = 50 μm approx

**Fig. 2:**
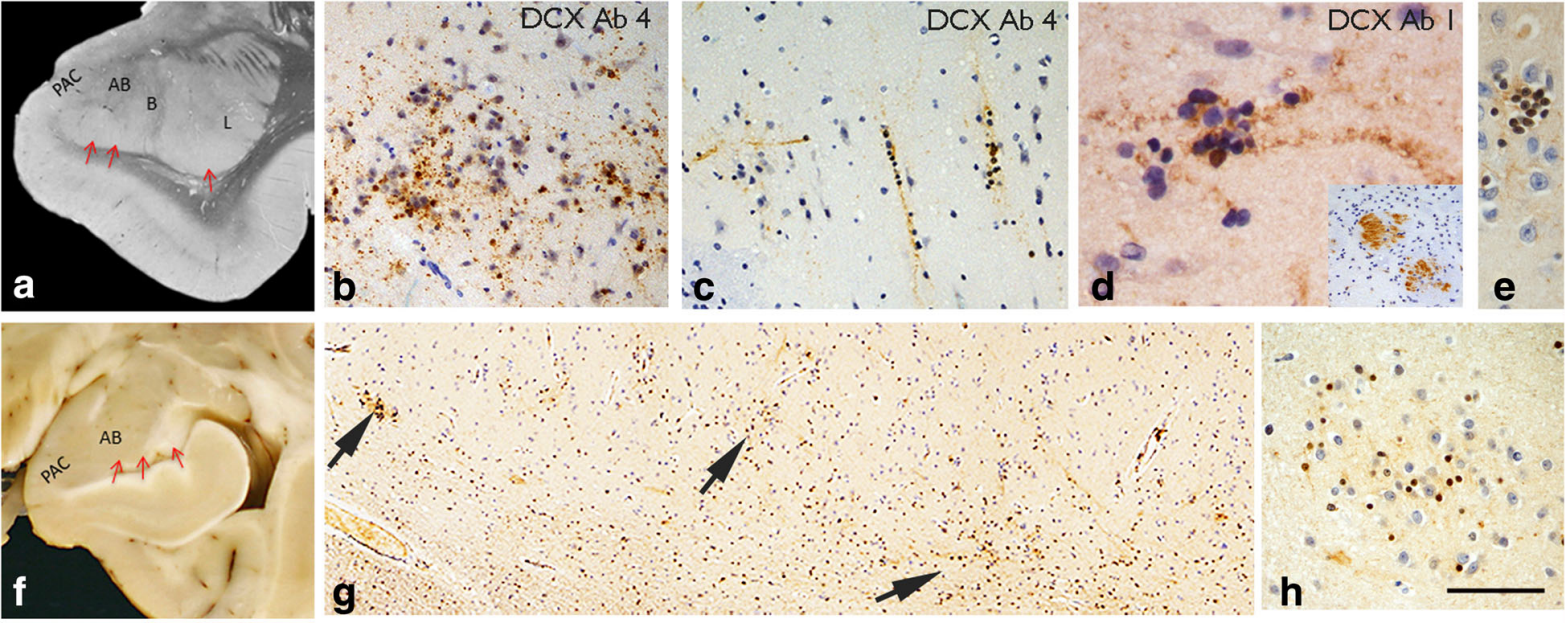
Amygdala and DCX expression. **a** Coronal level though caudal amygdala in a post mortem case indicating the location of the paralaminar nucleus with red arrows. PAC = peri-amygdala cortex, AB = accessory basal, B = basal and L = lateral nuclei. **b** DCX positive cells clusters and beaded fibres in the PAC of a surgical patient with TLE/HS; **c** In a further case, columns of DCX+ cells were seen in the PAC as well as horizontal processes. **d** Further surgical TLE/HS case with DCX Ab1 (see Table 2) with clusters of small immature cells with beaded processes and some with nuclear labelling (inset: shows coarser DCX+ bundles traversing the amygdala in a surgical case). **e** Clusters of small densely labelled immature DCX+ cells and processes in the amygdala in post mortem samples of paralaminar nucleus. **f** Post mortem caudal amygdala indicating the location of the paralaminar nucleus above the ventricle. **g** The paralaminar nucleus shown at low magnification in DCX labelled section, with nests of positive cells and clusters of processes indicated (arrows) running along the border. **h** At higher magnification these clusters correspond to small DCX^+^ cells intermingled with DCX immunonegative-negative mature neurons. Bar in A, F = 800 μm approx.; G = 100 μm approx.; in B, C, E, H = 50 μm approx.; in D = 20 μm approx

Tissue was examined from six regions of the temporal lobe in the majority of adult surgical cases, including: (i) temporal neocortex (superior temporal gyrus to fusiform gyrus at 1 cm rostral to temporal pole) (Fig. 1a), (ii) temporal pole, (iii) mid-hippocampus body, (iii) pes hippocampus, (iv) parahippocampal gyrus (PHG) and (v) amygdala. As a standard anterior temporal lobectomy procedure was performed and a routine tissue handling and processing protocol was followed, the regions selected were anatomically comparable between cases. In surgical cases, the amygdala tissue was typically fragmented which limited identification of all subnuclei. In PM cases, coronal sections of the mid hippocampal body, adjacent temporal cortex and/or sections through the entire mid to caudal amygdala, including the paralaminar nuclei, were examined (Fig. 2a, Additional file 1: Table S1 for details).

### Immunohistochemistry

Immunochemistry for DCX was carried out on 5 μm thick formalin-fixed, paraffin-embedded sections. We trialed four DCX commercially-available antibodies on selected surgical and PM cases. All four antibodies were previously validated in publications on human tissues [9, 28, 34, 40, 46] (Table 2; DCX Ab1 to 4). All DCX antibodies showed immunopositive labelling, but DCX Ab1 (clone 4604, Cell signaling, Boston, USA) labelled a greater diversity of cell types, and more intense labelling was observed in both surgical and PM human brain tissues. Consequently, DCX Ab1 was used across all cases for the descriptive and quantitative analysis. Double label immunofluorescent studies were performed on all anatomical regions of five selected surgical cases (Additional file 1: Table S1); combinations included DCX Ab1 with different DCX antibodies, and DCX Ab1 with astroglial (GFAP, GFAP-∂), microglial (Iba1, HLA-DR, CD68), mature neuronal (NeuN), immature/stem cells (nestin, CD34, SOX2), oligodendroglia (OLIG2), NG2-cells and pericytes (PDGFRβ) [11, 16] and cell cycle markers (MCM2). Details of the immunostaining protocols are included in Table 2 and the Additional file 2.

**Table 2:** Immunohistochemistry panel

Antibody	Product code, Supplier	Dilution (method)	Target Epitope
**DCX** Ab1	4606, Cell Signaling Tech. Boston, US. *Used in [28]	1:250 (IHC, IF)	Amino acid sequence 40–70 and 350–410 of human DCX
DCX Ab2	AB18723, Abcam, Cambridge, UK. *Used in [34, 40]	1:4000 (IF)	AA 300 to the C-terminus of synthetic human DCX
DCX Ab3	SC-8066, Santa Cruz Biotech. Heidelberg, Germany. *Used in [11, 24, 27]	1:400 (IF)	C-terminus 365–402 of human DCX
DCX Ab4	AB2253, EMD Millipore, Watford UK. *Used in [45, 46]	1:1000 (IHC, IF)	C-terminus 350–365
Nestin	AB22035, Abcam, Cambridge, UK.	1:1000 (IHC, IF)	150 aa recombinant fragment from human nestin conjugated to GST
Nestin	AB105389, Abcam, Cambridge, UK.	1:100 (IF)	Synthetic peptide corresponding to the C terminus of Human Nestin.
Sox 2	AB5603, EMD Millipore, Hertfordshire, UK.	1:400 (IF)	KLH-conjugated linear peptide corresponding to a C-terminal region sequence of human Sox2
GFAP-∂	AB93251, Abcam, Cambridge, UK,	1:4000 (IF)	Synthetic peptide conjugated to KLH derived from within residues 350 to the C-terminus of Mouse GFAP ∂
GFAP	Z0334, DAKO, Cambridgeshire, UK.	1:1500 (IF)	GFAP
NeuN	MAB377, EMD Millipore, Hertfordshire, UK.	1:100 (IF)	Purified neuronal nuclei
Iba1	019–19,741, WAKO, Osaka, Japan.	1:6000 (IF)	Synthetic peptide corresponding to C-terminus of Iba1
CD68	AB783, Abcam, Cambridge, UK.	1:50 (IF)	Macrophages, microglia
CD34	IR632, DAKO, Cambridgeshire, UK.	1:25 (IF)	Endothelial cells
Olig 2	AB9610, EMD Millipore Hertfordshire, UK	1:200 (IF)	Recombinant mouse Olig-2
PDGFR-beta	AB32570, Abcam, Cambridge, UK.	1:1000 (IF)	Synthetic peptide within Human PDGF Receptor beta aa 1050 to the C-terminus
MCM2	610,700, BD biosciences, Oxford, UK.	1:900 (IF)	Human BM28 aa. 725–888

For all antibodies, sections were pre-treated in sodium citrate solution (pH 6.0) microwaved at 800 W for twelve minutes. All primary antibodies were incubated overnight at 4 °C, except for anti-Iba1, CD68 and GFAP which were incubated for an hour at room temperature, and anti-GFAP ∂ which was incubated for 48 h at 4 °C. *Previous studies using DCX in human tissue studies.

Abbreviations: *IHC* immunohistochemistry, *IF* immunofluorescence

### Quantitative and qualitative analysis

DCX-immuno-labelled cells (DCX^+^) at the boundaries of cortical layer I/II in the temporal lobe of all cases were quantified using Image pro plus (Media Cybernetics, Cambridge, UK). Sequential images were captured at × 40 using a Leica DBMR microscope along the entire length of layer I and II from the gyrus to the sulcus of the most inferior-mesial gyrus (fusiform gyrus (FG) or inferior temporal gyrus (ITG))(Fig. 1a). DCX^+^ cells of different morphologies were counted and expressed as cells per mm of gyrus length. DCX^+^ cell types in other anatomical regions were semi-quantified as: (0) absent, (+) rare/occasional, (++) moderate numbers, (+++) many cells. In PM cases where the entire coronal cross-section of the paralaminar nucleus was present, the presence of DCX^+^ cells was evaluated as: (0) absent, (+) single cluster, (++) 2–3 clusters, and (+++) > 4 clusters. In the adjacent peri-amygdala cortex (PAC; Fig. 2a) DCX in the superficial cortex was assessed as: (+) occasional single DCX^+^ cell, (++) several single cells or one cluster, (+++) 2–3 clusters, (++++) > 4 clusters.

Qualitative examinations of double-labelled sections were carried out with a Zeiss Axio Imager Z2, and confocal laser scanning microscope (LSM-Meta 710, Zeiss, Göttingen, Germany). The software, Zen 2012 blue lite version (Zeiss, Göttingen, Germany), was used to view z-stacks of confocal images, and to compose three-dimensional orthogonal projections.

### DCX mRNA analysis

Histology data was compared to DCX mRNA expression analyses from 83 surgical TLE cases (Table 1); in 16 of these cases there was evidence of temporal neocortical sclerosis (layer II/III neuronal loss and gliosis) in addition to hippocampal sclerosis [43]. TLE samples were obtained from the UCL Epilepsy Society Brain and tissue bank and compared to 73 PM non-epilepsy control tissues samples from the MRC Sudden Death Brain and Tissue Bank (Table 1). This data formed part of a separate study (Matarin et al., in preparation). We also compared this data to pre-existing expression array data; specifically DCX mRNA expression in ten different brain regions of non-epilepsy control cases including occipital, temporal, frontal, hippocampus, putamen, thalamus, substantia nigra, white matter, medulla and cerebellum as well as different life periods from embryonic to late adulthood [21].

For temporal lobe cases (patients and controls), RNA was isolated from the middle temporal gyrus (MTG) cortex and samples were randomly placed and hybridized to Affymetrix Exon 1.0 ST arrays as previously described [22]. Control tissue from other brain regions and different life periods were also originally analysed with Affymetrix Exon 1.0 ST arrays [21] and data files were downloaded from the NCBI Gene Expression Omnibus (accession GSE25219 and GSE46706). Quality control analyses included background correction, quantile normalization, log2 transformation and median polishing probe-set summarization. The effects of several methodological and biological covariates were tested for significance and was included in the linear regression or MANOVA models when significant. All gene expression data was analyzed with Affymetrix Power Tools software package and Partek Genomics Suite (Partek Incorporated, St. Louis, MO, USA) and R software.

### Clinical data

The age of onset of seizures, age at surgery, seizure types, laterality and outcomes at two years following surgery were obtained from the clinical records of epilepsy patients (Table 1 and Additional file 1: Table S1). Pre-operative memory function tests were carried out as part of the routine clinical assessment as previously described [44] and detailed in Additional file 2: Methods. Data on memory function was available in 15 adult surgical patients (Additional file 1: Table S1). Statistical analysis was carried out between pathology and clinical groups using non-parametric tests (including Kruskal-Wallis with post-hoc corrections and Spearman correlation) SPSS (IBM, California, version 22); *p* values of ≤0.05 were regarded as significant.

## Results

### Qualitative findings

#### DCX^+^ neuronal cell types

Neuronal-appearing unipolar (Fig. 1b), bipolar (Fig. 1c) and multipolar DCX^+^ cells (Fig. 1d) were identified mainly at the interface between layer I and II of the temporal cortex as single cells or more rarely in clusters or rows (Fig. 1d); they were observed in 18 of the 22 surgical cases studied and henceforth are collectively referred to as ‘layer II DCX^+^ cells’. The processes of multipolar tufted cells extended mainly perpendicularly to the cortical surface, projecting into layer I, where rarer horizontally-orientated DCX^+^ cells and processes could be observed. In all cases, layer II DCX+ cells intermingled with other small, intensely labelled DCX^+^ cells without processes in layer II (Fig. 1b) and occasionally with larger DCX^+^ cells with more overt neuronal morphology (Fig. 1b). Layer II DCX^+^ cells appeared more frequent along the ITG and FG compared to MTG in surgical TLE cases. Layer II DCX^+^ cells were also observed in the temporal pole of over half of the cases studied, but less frequently in the resected parahippocampal gyrus (PHG) and PM TLE and control cases. Rare large DCX^+^ neuronal cells were noted in the granule cell layer of the hippocampus in surgical cases (Fig. 1h).

#### DCX^+^ glial cell types

Ramified DCX^+^ cells with multiple, branching processes were visualized mainly with the DCX antibody DCX Ab1 (Fig. 1e). Ramified DCX^+^ cells were present in all regions of all TLE cases, with and without HS, and all PM cases and noted in the gray and white matter. In the hippocampus, ramified DCX^+^ cells were particularly prominent in the hilus and subgranular zone of the dentate gyrus, extending through the granule cell layer. Occasional clusters or columns of DCX^+^ cells were seen (Fig. 1g) and proximity to CA1 pyramidal neurones. There were also present in the pes hippocampus and PHG in all cortical layers and white matter. Ramified cells were morphologically reminiscent of microglia and NG2^+^ cells as well as immature migrating neurons in the developing fetal brain in the periventricular zone (Fig. 1j). Occasional cells were also noted alongside vessels. In addition, a population of small round DCX^+^ oligo-like cells, without cytoplasmic processes, were scattered in white matter and cortex, visualized with all DCX antibodies; similar cells were often seen in a satellite position adjacent to neurons (Fig. 1i), particularly in deep cortical layers, as previously reported [40].

#### DCX^+^ cells in amygdala

##### Surgical cases

The peri-amygdala cortex (PAC) and paralaminar/periventricular nuclei could be anatomically identified in surgical cases, but were incompletely represented. All DCX antibodies labelled clusters of small DCX^+^ cells, mainly in the superficial cortex of the PAC (Fig. 2b-d). These DCX^+^ cells were sometimes arranged in horizontal or vertical columns, with beaded linear processes (Fig. 2c). Fragments of amygdalar nuclei with a ventricular border (paralaminar nuclei) showed aggregates of small, intensely-labelled DCX^+^ cells and fibres intermingled with DCX-negative, mature neurons. Coarse fibre tracts and bundles of DCX^+^ processes were also occasionally noted in the amygdala of all surgical cases (Fig. 2d, inset). In addition, ramified DCX^+^ cells with cytoplasmic labelling, as observed in other regions, were widespread. **PM cases:** Variable labelling of small DCX^+^ cell aggregates in the paralaminar nucleus of the amygdala was noted, particularly along the ventricle wall in the caudal amygdala (Fig. 2f, g). In these regions, clusters of small immature DCX^+^ nuclei were intermingled with larger and intermediate-sized DCX-negative neurones. (Fig. 2h). Some DCX^+^ cells were associated with fine beaded bipolar processes Fig. 2e). In the PAC, scattered small immature DCX^+^ cells with processes were noted, occasionally in clusters, mainly at the interface between layer I and II in either horizontal or perpendicular arrangements, as in the surgical cases.

#### Double labelling studies and comparison of different DCX antibody labelling

##### DCX antibodies

**Fig. 3:**
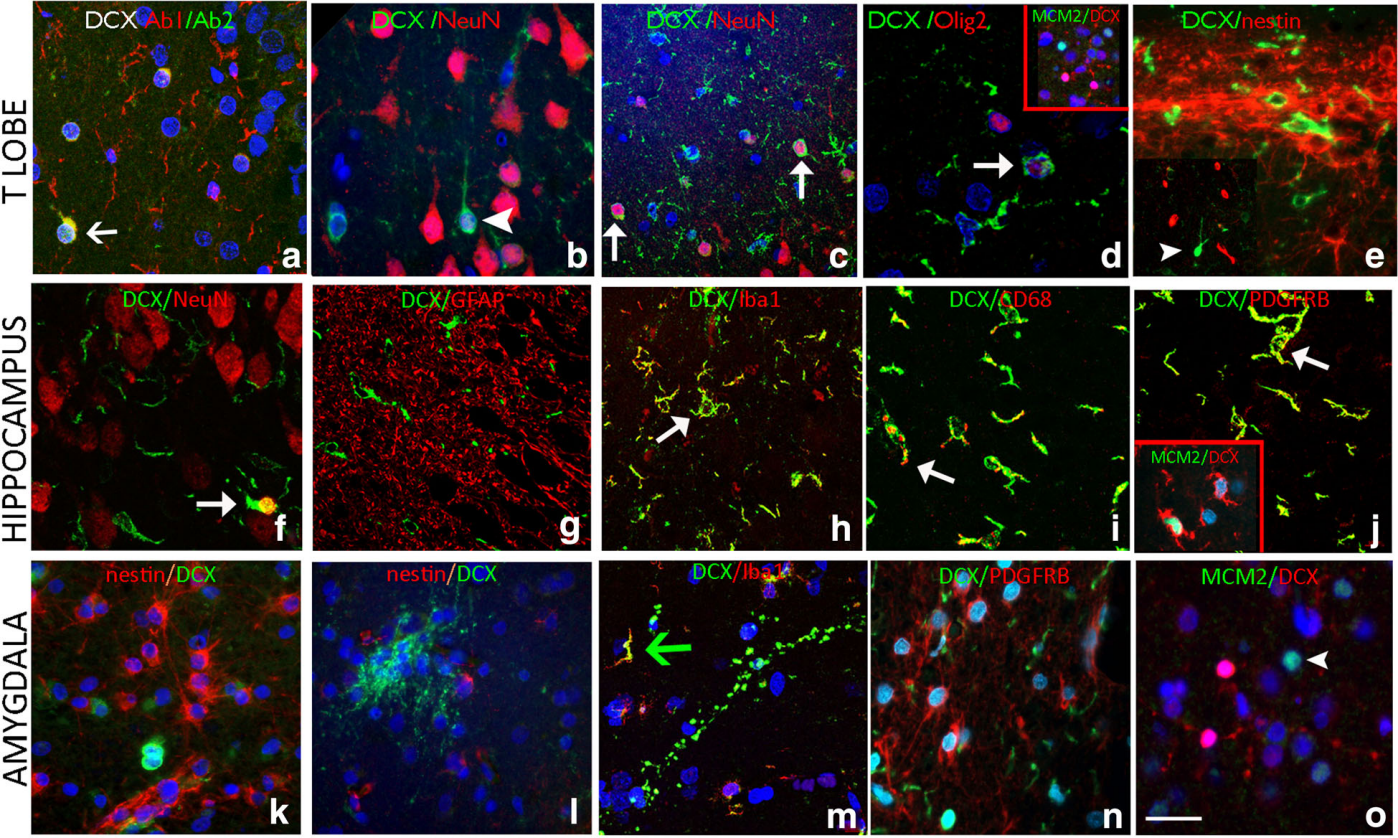
Characterizing DCX positive cells in the temporal cortex, hippocampus and amygdala of surgical patients with epilepsy, and comparison of commercial antibodies. In all panels, the arrowheads indicate single labelled cells, while arrows point to double labelled cells. Confocal images are merged projections of 5 to 7 images acquired in a z-stack. **a**. The immunoreactivity of two different commercially-available anti-Dcx antibodies. Both DCX Ab2 AB18723 (Abcam, Cambridgeshire, UK) and DCX Ab 1 #4606 (Cell Signaling Technology, Inc. MA, USA) labelled small cells in the hippocampal granule cell layer (GCL) of a patient with epilepsy and HS Type 1 (arrow) (Table 2). DCX Ab1 labelled more cells overall than DCX Ab2. ***DCX+ positive cells in the remaining figures were labelled using DCX Ab1.*** **Temporal Cortex Layer I/II: b**. NeuN expression was not frequently observed in small DCX+ cells located in layer II of the temporal lobe cortex (arrowhead). **c**. In another case, DCX/NeuN positive cells were more frequently observed in the superficial temporal cortex than in the temporal pole (arrows). **d**. DCX positive cells expressing Olig2 in the nucleus (arrow) were noted in layer 1 of an epilepsy case (arrow), but MCM2/DCX+ cells were not observed (inset). **e**. Nestin+ glial fibres were observed in the subpial layer and layer I and II, but did not co-localise with DCX expression. **Hippocampus: f**. In general there were rare DCX/NeuN colocalised cells in the dentate gyrus; in this image there is a rare co-localised cell (arrow). **g**. GFAP showed dense labelling of astroglial process in hippocampal regions but no co-localisation with DCX was noted. **h**. Labelling with Iba1 highlighted mature microglial cell types, with ramified processes, particularly in the subgranular zone as shown, and many co-expressed DCX (arrow); **i** Co-labelled CD68/DCX+ cells were also observed (arrow). **j**. PDGFRβ was expressed in multipolar cells in the hippocampus and temporal lobe in addition to pericytes; focal co-labelling with DCX was noted in some cells (arrow); Inset show a DCX+ ramified cell expressing MCM2 in the nucleus. **Amygdala. k**. Distinct populations or clusters of nestin+ or DCX+ cells in the amygdala periventricular nuclei; **l**. In another area, nests of DCX+ processes were also distinct from nestin-expressing cells. **m**. In the amygdala, although DCX+ processes were largely Iba1 negative, Iba1/DCX+ ramified DCX+ cells were observed (arrow). **n**. PDGFRβ and DCX in the amygdala showing distinct populations of small cells. **o**. DCX in the amygdala were mainly MCM2 negative. Bar is equivalent to 20 μm. Individual channels for immunofluorescence images are shown in Additional file 4: Figure S2

DCX Ab1 showed the most extensive labelling of cells and processes in different regions but co-localization was confirmed in a proportion of small cells with the three other different DCX antibodies (Table 2, Fig. 3a). **Temporal neocortex:** In layer II of the temporal lobe, DCX^+^ tufted cells showed only rare co-localisation with mature neuronal marker NeuN (Fig. 3b, c). There was no cellular co-expression of DCX with mature glial marker GFAP, immature stem cell markers nestin or GFAP∂, which both showed labelling of the sub-pial band of astrocytes (Fig. 3e). There were a few DCX^+^/Sox2^+^ cells noted. There were no CD34^+^ neuroglial cells in any of the surgical epilepsy cases. A minor proportion of small oligo-like DCX+ cells in the white matter co-expressed OLIG2 (Fig. 3d); in contrast, MCM2/DCX^+^ cells were not observed (Fig. 3d inset). There was extensive co-localisation between ramified DCX^+^ cells and Iba1, CD68 and, to a lesser extent, PDGFRβ. **Hippocampus body and pes:** Very rare DCX^+^/NeuN^+^ cells were observed in the granule cell layer (Fig. 3f). We did not observe any co-expression between GFAP, GFAP∂ and nestin with DCX and these markers highlighted distinct cell populations in all regions (Fig. 3g). There was, however, prominent co-localization of ramified, multipolar DCX+ cells with Iba1 (Fig. 3h) and CD68 (Fig. 3i), particularly in the granule cell layer region. In addition, co-expression of DCX with PDGFRβ was evident in some small branching cells as well as MCM2 (Fig. 3j). Occasional double-labelling of small oligo-like cells with DCX and OLIG2 as well as SOX2 was noted in hippocampal regions but not with CD34. **Amygdala:** Although DCX^+^ and nestin-expressing cells were observed in similar anatomical regions, particularly in the paralaminar nucleus, they highlighted distinct cell populations and processes (Fig. 3k, l). Multipolar, ramified DCX^+^ cells showed co-expression with Iba1 (Fig. 3m) and occasionally with PDGFRβ (Fig. 3n), but only in a proportion of cells. Cellular co-expression of DCX with NeuN, CD34, GFAP or MCM2 (fig. 3o) was not observed in the amygdala of the cases examined and there was rare co-expression of DCX with OLIG2 or SOX2.

#### Quantitative analysis

Based on the above findings our hypothesis was that quantitative analysis would confirm greater densities in the number of DCX^+^ cells in epilepsy than control groups. In the **temporal lobe** we confirmed higher linear densities for all morphological DCX^+^ cell types in surgical epilepsy cases compared to PM epilepsy cases and non-epilepsy controls (Fig. 1k) with statistically significant differences noted for ramified DCX^+^ cell populations (*p* < 0.0001). We also investigated their relationship with patient age and cortical region. The linear density of layer II cells compared to other DCX^+^ cell types showed an inverse correlation with age for all cases (surgical and PM) (*p* = 0.001) (Fig. 1l) as well as for surgical cases alone (*p* = 0.016) and for age of seizure onset (*p* = 0.035). There were significantly higher linear densities of tufted DCX^+^ cells in the FG compared to the ITG (*p* = 0.004) but not for other DCX^+^ cells types. We also hypothesized if there was a relationship between DCX^+^ linear densities and the presence of HS but did not confirm this. Similarly, we considered if DCX cell densities related to clinical parameters; there was no significant difference in DCX^+^ cells in relation to seizure types (focal or generalized seizures), history of aura (Fig. 1m) or if patients were seizure free (or not) two years following surgery. We noted an association for increased ramified DCX^+^ in cortical layer II in patients with severe verbal pre-operative memory deficit (Additional file 3: Figure S1; *p* = 0.027). In other regions **(hippocampus, pes hippocampus, PHG and temporal pole and amygdala),** semi-quantitative analysis of DCX^+^ ramified and small cell types in surgical cases did not show any significant differences between cases with HS or without HS, relationship to age at surgery, age of seizure onset, seizure types or outcome at 2 years. In PM cases, there were no significant differences in DCX populations in amygdala and PAC between epilepsy controls and controls; there was a negative correlation between DCX^+^ cells in the PAC and age over all PM cases (*p* = 0.04).

#### mRNA expression

**Fig. 4:**
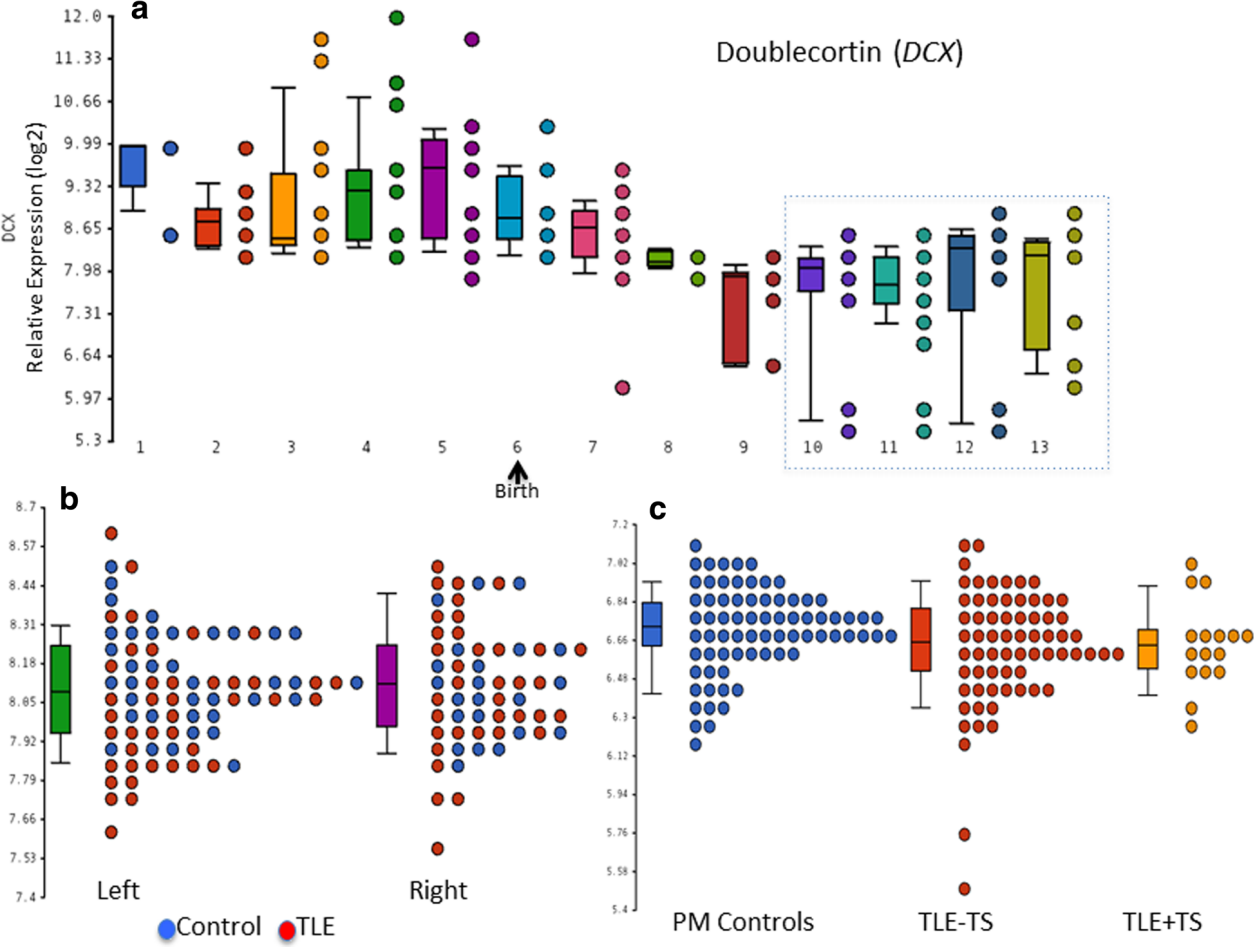
DCX mRNA expression data. **a** Significant DCX temporally differential expression between periods on temporal cortex (*p* value: 4.5e-^113^) [21]. Periods 1: Embryonic, 2: Early Fetal, 3: Early midfetal 4: Late midfetal- 5 Late fetal 6 Neonatal and early Infancy 7 Late infancy 8 Early childhood 9 Middle and Late adulthood 10:Adolescence 11: Young adulthood 12: Middle adulthood 13: Late adulthood **b** DCX expression on right (R) and left (L) temporal cortex on patients with temporal lobe epilepsy (TLE) [22] and controls [21] after correcting for batch differences. Periods 10, 11 and 12 **c)** DCX mRNA expression data from 83 TLE (divided into cases with or without temporal lobe sclerosis (TS) (neuronal loss from the superficial neocortex in addition to hippocampal sclerosis [43]) and controls from the MRC brain bank, Edinburgh, after correcting for batch, age, gender and RIN differences. Periods 10 to 13

From published data [21] *DCX* mRNA was expressed in all brain regions tested and exhibited temporal and regional significant differential expression (False Discovery Rate 5%). There was higher *DCX* mRNA expression in temporal, frontal and occipital regions and lower in cerebellum. DCX expression in the temporal cortex was higher in periods before and early birth (embryonic, fetal and early infancy) than in periods following the early infancy (late infancy, childhood, adolescence and adulthood) (Fig. 4a). We observed no significant differences in *DCX* expression in the temporal cortex between left and right hemispheres in controls [21] or TLE patients (adolescence and young and middle adulthood periods) (Fig. 4b). We did not find significant differences between patients with and without temporal lobe sclerosis and neither between patients with epilepsy and the 2 PM control groups (Fig. 4c) (For all *p* values > 0.05 and fold change < 1.2).

## Discussion

DCX is currently widely used as developmental marker for post-mitotic, immature neurons in the brain and for persistent neurogenesis in adulthood [14, 37]. Our study highlights the varied morphologies of DCX^+^ cells in adult TLE with ongoing expression supported by a parallel gene expression study. Tangled DCX^+^ cells in layer II of the temporal cortex did not express nestin, NeuN or MCM2, declined in numbers with age and did not significantly associate with clinical seizure semiology, memory dysfunction or the presence of HS. Amygdala DCX+ immature neuronal populations were primarily located in the paralaminar nucleus, but their number and morphology were not different between cases and controls. We also identified a subset of ramified and small cells with DCX antibodies, representing a range of glial cell types and supporting non-neuronal DCX expression; these were increased in number in the neocortex in epilepsy, associated with memory impairment and may represent reactive populations.

### Temporal cortical DCX^+^ cells in epilepsy

The presence of persistent, immature, DCX^+^ neocortical cells in layer II, primarily in the temporal lobe and piriform cortex, has been recognized in a range of adult mammalian species since 2001 (reviewed in [6]). They are less studied in humans and their precise physiological function remains elusive. They have been variable termed ‘tangled and tufted’ cells or ‘semilunar/transitional’ neurons, co-express PSA-NCAM or other immature neuronal markers but reported to be mainly negative with mature neuronal marker NeuN. Although the number of DCX^+^ cortical cells decline with age, they are considered to endow the cortex with enhanced synaptic plasticity [23] and roles in olfactory learning and memory have been postulated [6]. In mammals they are not considered to be newly generated in adulthood, but to arise during development from migrating cells of the lateral cortical stream [2]. A persistent *temporal lobe migratory stream* has also been recently described in adult mammals, extending from the temporal pole of the lateral ventricle to the piriform cortex and amygdala [14] and we have also recently reported evidence for vestiges of a similar migratory stream in the human adult mesial temporal lobe [27]. In TLE an increased plexus of PSA-NCAM-positive neurons was reported in layer II of the entorhinal cortex [30] and increased DCX^+^ cells were noted in the temporal neocortex in epilepsy compared to controls with evidence for some maturation with NeuN expression, yet maintaining a persistent immature phenotype (98% co-expressing PSA-NCAM and 82% Tuj1) [28]. Similar tangled DCX^+^ cells in layer II were also prominent in young patients with FCD type Ia [40].

In the current series, layer II DCX+ temporal neocortical cells showed infrequent NeuN expression, in keeping with some previous studies [15, 40]; the additional lack of expression of the stem cell marker nestin could support their intermediate differentiation between progenitor cells and mature neurons [27]. We confirmed their anatomical distribution, with increased density in more mesial temporal neocortex, as suggested in previous reports [6, 28]. They were not prominent in the parahippocampal gyrus cortex however and we also observed a significant decline in the number of tangled cells with age. In our recent study of TLE/HS patients aged over 50 years with early Alzheimer-like pathology in temporal lobe resections, we reported a lack of tau accumulation in layer II DCX+ cells, suggesting their resistance to age-related neurodegeneration [42], as also supported by experimental models [52]. We have shown in this study that the number of tangled neurons is not increased in epilepsy patients compared to controls, nor is the number related to clinical olfactory/sensory auras, memory dysfunction the presence of hippocampal sclerosis or post-surgical outcome. In a parallel gene expression study from patients with TLE/HS there were no significant differences in patients with additional temporal lobe superficial cortical neuronal loss and gliosis (also called temporal lobe sclerosis) [43] from those with normal/preserved neocortex. These findings could suggest that layer II DCX+ cell types are unlikely to be clinical or pathologically relevant to functional and acquired pathologies in temporal lobe epilepsy.

### Amygdala and DCX^+^ populations

The amygdala can initiate seizures in TLE and volume changes, including enlargement are recognized in TLE in addition to gliosis [3]. During development, migrating DCX+ cells in the lateral cortical stream give rise to neuronal and glial populations in the amygdala [2]; persisting DCX+ cells in adulthood have been reported in the primate amygdala and PAC, forming chains of cells, a proportion of which co-express NeuN, their number declining with age [52]. Similar cell types were also confirmed in the human amygdala [29], in particular the paralaminar nucleus of the amygdala, where abundant rests of immature DCX+ neurons were shown in one study [10]. The paralaminar nucleus, which sits along the ventricle wall, is composed of nodules of small primitive appearing cells, and is interconnected with other amygdala nuclei receiving high serotonergic input. Its precise functions are uncertain [10]. In the current study, DCX^+^ immature cells, primarily in the paralaminar nucleus and PAC, were confirmed to exist in the amygdala, in both surgical tissue as well as PM cases. Diminished populations were associated with increasing age but the cells did not show atypical morphology or altered numbers in epilepsy or associate with the presence of hippocampal sclerosis. Nevertheless, application of DCX^+^ may be helpful in clinical practice to enable their anatomical identification and avoid over-interpretation as small malformations or hamartias, described in surgical resections in TLE [17].

### DCX – A reliable marker of neurogenesis in the mature human brain?

There is increasing awareness of DCX expression in non-neuronal cells types. DCX-expressing astroglial cells have been noted in adult post mortem tissues in patients with epilepsy and controls [46], in balloon cells in FCD IIB and multipolar astroglial like cells in temporal lobe sclerosis [40]. DCX^+^ stellate cells have been reported in the vicinity of acute infarcts with co-expression of astroglial lineage markers (GFAP, S100) rather than microglial or mature neuronal markers as NeuN [25]. In another study however, DCX^+^ ‘rod’ cells in the infant hippocampal subgranular zone region, that morphologically resembled microglia, lacked CD68 and HLADR expression and were argued to represent neural progenitor cell types [34]. In adult mammals, DCX^+^ cells are reported to be enriched in the subgranular zone of the hippocampus, and are described as ‘ramified’, although exhibiting more overt neuronal morphology with apical dendrites [14, 36]. Experimental tracing studies of DCX^+^ cell fate during temporal lobe development show that 95% give rise to TBR1-positive pyramidal neurons but a small proportion differentiate to GFAP astrocytes (3.4%) and a further population of small bipolar cells with sparsely branching processes are of uncertain lineage (1.7%) [2]. Single cell transcriptomics of isolated DCX^+^ populations has also provided evidence of cell subsets enriched for divergent pathways, including astrocytic and myelinating oligodendroglial fates [15]. Identification of DCX expression in oligodendroglial precursor cells (OPC) was proposed to reflect cell migration rather than neuronal differentiation [7] and DCX expression is also speculated to have a role in glioblastoma infiltration [1].

Our current study adds further support to DCX expression in non-neuronal cell types. We noted frequent ramified, multipolar DCX^+^ cells and oligo-like cells without processes in many regions, including the SGZ, and these were significantly increased in surgical TLE compared to controls. These were observed mainly with one DCX antibody although overlap of expression was noted in a proportion of small cells with other commercial DCX antibodies, suggesting different sensitivities. Although DCX^+^ ramified cells in the dentate gyrus resembled cells in the developing human sub-ventricular zone, they lacked the typical unipolar or bipolar morphology [36]. Furthermore, we confirmed that a proportion of ramified DCX^+^ cells co-expressed microglial markers, as well as PDGFRβ, supporting non-neuronal lineages. Significantly more ramified DCX^+^ cells were noted in surgical epilepsy cases in the temporal cortex compared to controls. However there were no differences between epilepsy cases with or without hippocampal sclerosis. These different observations suggest enhanced DCX expression in reactive glial cells types in epilepsy could be independent of underlying pathology.

The observation of DCX in microglia was an unexpected finding. Microglial cells are dynamic, motile cells which show changes in morphology in response to brain activity and injury, with increasingly recognized diverse and complex roles [8, 32] including neurodevelopment as well as epilepsy [13]. Understanding of these cells has advanced with proteomic and transcriptomic analysis of isolated microglia, aiming to identify specific proteins more abundantly expressed, as candidate lineage and activation markers [12, 38, 48, 53] (see https://omictools.com/glia-open-access-database-tool). Although proteomics and gene expression studies have suggested that DCX is not expressed in microglia at significant levels [18–20, 38, 50, 53], differential gene expression studies do show DCX is dependent on their activation state. For example, one study using laser single cell capture and gene expression of microglial cell types of different morphology showed that DCX was highly enriched in activated amoeboid microglia and confirmed by immunohistochemistry and PCR of a murine microglial cell line [35]. Recent studies have also highlighted expression of putative immature neuronal markers in reactive cell types, including microglia in human slice cultures [47].

Intriguingly we also noted PDGFRβ^+^ co-expression in some of the ramified DCX^+^ cells in TLE tissues; DCX expression has been previously shown in pericytes [53]. Although PDGFRβ^+^ is widely recognized as a CNS pericyte marker [41] it is also expressed in OPC/NG2 parenchymal glial cell types, which are reactive to seizures [16, 26, 31, 39]. We also noted occasional DCX^+^ small cells to co-express OLIG2 in keeping with reported low DCX expression in OPC lineages [7, 53]. In our parallel gene expression study from TLE cortex, DCX expression was confirmed in adult temporal cortex and, although expression was not significantly increased in TLE compared to controls, more detailed single cell analysis is warranted to further investigate differential cellular expression in epilepsy. Our findings therefore support ongoing DCX expression in adults; expression in non-neuronal cells, particularly in adults undergoing surgery for refractory epilepsy, may suggest aberrant upregulation under pathological conditions, including seizures.

### Study limitations

There are several limitations to the study. The ages in the epilepsy and control groups for the histology were not precisely matched as in the mRNA study and although ramified DCX^+^ cell densities did not show a relationship with age, we cannot exclude that this may have had an effect. The PM cases with epilepsy and HS did not all have syndromic TLE and limited memory tests were available. The gene expression data on MTG was obtained on homogenized cortex from all cases rather than micro dissected cortical layers or cells and was not carried out on the ITG or FG where greater numbers of DCX^+^ cells are observed. We did not have complete representation of the FG in the surgical specimens, compared to the PM cases in the analysis of Layer II DCX^+^ cells and in the amygdala surgical specimens were fragmentation which limits analysis of the entire nucleus.

## Conclusions

DCX identifies a range of morphological cell types in temporal lobe epilepsy, including immature populations in the superficial cortex and amygdala that decline with age but may not be specifically relevant to the epilepsy or local pathology. We observed DCX^+^ reactive glial cell types, including microglial lineages with some evidence for increased numbers in epilepsy tissue. This suggests that DCX not only is expressed in residual immature neuronal cell types, but may have a role in brain responses to seizure injury.

## Additional files

Additional file 1: **Table S1.** Detail of each case used in the study including clinical and psychometric data and the type of study carried out (DOCX 29 kb)

Additional file 2: Supplementary Methods (DOCX 18 kb)

Additional file 3: **Figure S1.** Bar graphs of the relationship between pre-operative memory function and DCX+ cells in the superficial temporal lobe. **A**. There was an association between severe memory deficit and increased ramified type DCX+ cells (*p* = 0.027). However the number of patients in this series is small and requires validation with a larger cohort. **B**. There was no association between the number of tufted DCX+ neuronal cells in layer II of the temporal cortex and pre-operative memory function. (JPG 181 kb)

Additional file 4: **Figure S2.** Split channels for DCX double labelling as indicated in temporal lobe, hippocampus and amygdala for images shown in Fig. 3. (TIF 15727 kb)

## Abbreviations

DCX: Doublecortin
FG: Fusiform gyrus
HS: Hippocampal sclerosis
ILAE: International league against epilepsy
ITG: Inferior temporal gyrus
MTG: Middle temporal gyrus
OPC: Oligodendroglial precursor cells
PAC: Perimagydala cortex
PHG: Parahippocampal gyrus
PM: Post mortem
TLE: Temporal lobe epilepsy
