# Supplementary material for: Doublecortin-expressing cell types in temporal lobe epilepsy
Source: Acta Neuropathol Commun. 2018 Jul 13;6:60. doi: 10.1186/s40478-018-0566-5 (PMC6045867; doi:10.1186/s40478-018-0566-5)
Supplement: Supplementary file 1 — Table S1. Detail of each case used in the study including clinical and psychometric data and the type of study carried out (DOCX 29 kb) [file 40478_2018_566_MOESM1_ESM.docx]

| **GROUP** | **Case number** | **AGE OF ONSET OF EPILEPSY (Years)** | **AGE AT SURGERY**  **(Years)** | **GENDER** | **ILAE HS type IN HIPPOCAMPUS BODY** | **Seizure type / syndrome**  **/ Aura** | **ILAE TYPE IN PES** | **ABNORMAL PRE-OPERATIVE PSYCHOMETRY**  **GRADED NAMING TEST, VERBAL AND VISUAL MEMORY DYSFUNCTION**  **(MODERATE OR SEVERE)~** | **REGIONS USED IN THIS STUDY**  **(* cases used in IF study in addition to IHCq)** | **ADDITIONAL PATHOLOGY** |
| --- | --- | --- | --- | --- | --- | --- | --- | --- | --- | --- |
| ADULT MTLE/HS TYPE 1 | S1 | 6 | 42 | F | TYPE 1 | PS, CPS, GS  Aura+ | TYPE 1 | Severe deficit GNT | HIPPOCAMPUS BODY, PES, AMYGDALA, PHG, TEMPORAL LOBE AND POLE |  |
|  | S2 | 17 | 36 | M | TYPE 1 | CPS, GS  Aura- | TYPE 1 | No deficit | HIPPOCAMPUS BODY, PES, AMYGDALA, PHG, TEMPORAL LOBE AND POLE* |  |
|  | S3 | 11 | 34 | M | TYPE 1 | PS, CPS, GS  Aura + | TYPE 1 | No deficit | HIPPOCAMPUS BODY, PES, AMYGDALA, PHG, TEMPORAL LOBE AND POLE* |  |
|  | S4 | 10 | 29 | F | TYPE 1 | PS, CPS  Aura + | TYPE 3 | No data available | HIPPOCAMPUS BODY, PES, AMYGDALA, PHG, TEMPORAL LOBE AND POLE* |  |
|  | S5 | 2 | 39 | M | TYPE 1 | PS, CPS, GS  Aura + | TYPE 1 | Moderate deficit GNT and visual memory  Severe deficit verbal memory | HIPPOCAMPUS BODY, PES, AMYGDALA, PHG, TEMPORAL LOBE AND POLE |  |
|  | S6 | 3 | 54 | F | TYPE 1 | CPS, GS  Aura + | INCOMPLETE | Moderate deficit GNT and verbal memory  Severe deficit visual memory | HIPPOCAMPUS BODY, PES, AMYGDALA, PHG, TEMPORAL LOBE AND POLE |  |
|  | S7 | 33 | 45 | F | TYPE 1 | CPS, GS  Aura - | INCOMPLETE | No data available | HIPPOCAMPUS BODY, PES, AMYGDALA, PHG, TEMPORAL LOBE AND POLE* | MILD FCDIIIA (BLOCK 1C) |
|  | S8 | 14 | 22 | F | TYPE 1 | CPS, GS  Aura + | TYPE 1 | Moderate deficit GNT  Severe deficit verbal and visual memory | HIPPOCAMPUS BODY, PES, AMYGDALA, PHG, TEMPORAL LOBE AND POLE* | PREVIOUS INTRACRANIAL ELECTRODES |
|  | S9 | 35 | 51 | M | TYPE 1 | CPS, GS  Aura - | TYPE 3 | No deficit | HIPPOCAMPUS BODY, PES, AMYGDALA, PHG, TEMPORAL LOBE AND POLE |  |
|  | S10 | 31 | 45 | M | TYPE 1 | CPS. GS  Aura - | TYPE 3 | Severe deficit GNT | HIPPOCAMPUS BODY, PES, AMYGDALA, PHG, TEMPORAL LOBE AND POLE |  |
|  | S11 | 3 | 53 | F | TYPE 1 | CPS, GS  Aura + | INCOMPLETE | Moderate deficit in visual memory | HIPPOCAMPUS BODY, PES, AMYGDALA, PHG, TEMPORAL LOBE AND POLE |  |
|  | S12 | 6 | 33 | M | TYPE 1 | CPS, GS  Aura - | TYPE 3 | Moderate deficit in verbal memory  Severe deficit in visual memory | HIPPOCAMPUS BODY, PES, AMYGDALA, PHG, TEMPORAL LOBE AND POLE |  |
|  | S13 | 14 | 54 | F | TYPE 1 | CPS, GS  Aura + | TYPE 1 |  | HIPPOCAMPUS BODY, PES, AMYGDALA, PHG, TEMPORAL LOBE AND POLE | VARIABIITY IN PATTERN OF HS IN BODY |
| PAEDIATRIC TYPE 1 HS | S14 | UK | 8 | M | TYPE 1 | CPS | NOT EXAMINED | Not available | HIPPOCAMPUS BODY,  TEMPORAL LOBE |  |
|  | S15 | UK | 14 | F | TYPE 1 | AS, SGS | NOT EXAMONED | Not available | HIPPOCAMPUS BODY,  TEMPORAL LOBE |  |
|  | S16 | UK | 11 | F | TYPE 1 | CPS | NOT EXAMINED | Not available | HIPPOCAMPUS BODY,  TEMPORAL LOBE |  |
|  | S17 | UK | 15 | M | TYPE 1 | CPS, SGS | NOT EXAMINED | Not available | HIPPOCAMPUS BODY,  TEMPORAL LOBE |  |
|  | S18 | UK | 13 | M | TYPE 1 | CPS, SGS | NOT EXAMINED | Not available | HIPPOCAMPUS BODY,  TEMPORAL LOBE |  |
| No hippocampal sclerosis in body | S19 | 20 | 35 | M | NO HS | CPS, GS  Aura - | INCOMPLETE | Moderate deficit GNT and verbal memory  Severe deficit visual memory | HIPPOCAMPUS BODY, PES, AMYGDALA, PHG, TEMPORAL LOBE AND POLE | PREVIOUS INTRACRANIAL ELECTRODES |
|  | S20 | 19 | 24 | M | NO HS | PS, CPS, GS  Aura + | NO HS | No deficit | HIPPOCAMPUS BODY, PES, AMYGDALA, PHG, TEMPORAL LOBE AND POLE | MILD MCD IN TEMPORAL LOBE |
|  | S21 | 9 | 28 | F | NO HS | PS, CPS  Aura + | INCOMPLETE | No deficit | HIPPOCAMPUS BODY, PES, AMYGDALA, PHG, TEMPORAL LOBE AND POLE | POSSIBLE LESION IN AMYGDALA ON MRI - NOT CONFIRMED IN PATHOLOGY |
|  | S22 | 22 | 24 | M | NO HS | CPS, GS  Aura + | TYPE 3 | No data available | HIPPOCAMPUS BODY, PES, AMYGDALA, PHG, TEMPORAL LOBE AND POLE | MRI LESION IN AMYGDALA NOT CONFIRMED |
|  | S23 | 15 | 30 | F | NO HS | CPS, GS  Aura - | TYPE 1 | Moderate deficit in verbal memory | HIPPOCAMPUS BODY, PES, AMYGDALA, PHG, TEMPORAL LOBE AND POLE | PREVIOUS INTRACRANIAL ELECTRODES |
|  | S24 | 15 | 31 | M | NO HS | PS, CPS, GS  Aura + | NO HS | Severe deficit in GNT | HIPPOCAMPUS BODY, PES, AMYGDALA, PHG, TEMPORAL LOBE AND POLE |  |
|  | **Case number** | **AGE OF ONSET OF EPILEPSY (Years)** | **AGE AT DEATH (Years)** | **GENDER** | **ILAE HS type IN BODY** | **Seizure type / syndrome** | **NEUROPATHOLOGYH AT PM**  **(IN ADDITION TO ANY HS)** | | **REGIONS USED IN THIS STUDY** | **CAUSE OF DEATH** |
| EPILEPSY POST MORTEMS | EPM1 | 4 | 72 | M | NO HS | FS, CPS, GS  SYNDROME : SFE | SUBACUTE INFARCT IN POSTERIOR CEREBEAL ARTERY TERITORY | | HIPPOCAMPUS BODY (BOTH SIDES), TEMPORAL LOBE | CVD |
|  | EPM2 | 4 | 26 | M | TYPE 1  BILATERAL | AbS, GTCS  SYNDROME : SFE | OLD INFARCT IN MIDDLE CEREBRAL ARTERY TERRITORY | | HIPPOCAMPUS BODY (BOTH SIDES), TEMPORAL LOBE | CONGENITAL HEART DISEASE |
|  | EPM3 | childhood | 47 | F | TYPE 1  BILATERAL | GTCS  SYNDROME : U/C | SMALL CORTICAL INFARCTS  TELANGIECTASIA | | HIPPOCAMPUS BODY (BOTH SIDES), TEMPORAL LOBE | CARDIAC DISEASE |
|  | EPM4 | 10 MONTHS | 71 | F | TYPE 1  BILATERAL | GTCS  SYNDROME : U/C | CEREBELLAR ATROPHY  AD pathology (Braak stage I/II) | | HIPPOCAMPUS BODY (BOTH SIDES), TEMPORAL LOBE | PE |
|  | EPM5 |  | 75 | M | NO HS |  | CEREBELLAR ATROPHY  LEWY BODY DISEASE | | HIPPOCAMPUS BODY TEMPORAL LOBE | bronchopneumonia |
|  | EPM6 |  | 27 | M | NO HS |  | PATHOLOGY NEGATIVE | | AMYGDALA | SUDEP |
|  | EPM7 |  | 41 | F | NO HS |  | PATHOLOGY NEGATIVE | | AMYGDALA | SUDEP |
|  | EPM8 |  | 34 | M | NO HS |  | AMYGDALA GLIOSIS | | AMYGDALA | SUDEP |
|  | EPM9 |  | 20 | F | NO HS |  | MILD CEREBELALR ATROPHY, OLD VENTRICULITIS | | AMYGDALA | SUDEP |
|  | EPM10 |  | 52 | M | NO HS |  | ULEGYRIA | | AMYGDALA | SUDEP |
|  | EPM11 |  | 58 | M | TYPE 1 HS  SIDE | TLE | RIGHT AMYG GLIOSIS | | AMYGDALA | PROB SUDEP |
|  | EPM12 |  | 56 | M | TYPE 1 HS  RIGHT |  | NONE | | AMYGDALA | UNKNOWN - HEAD ONLY |
|  | EPM13 |  | 69 | F | TYPE 1 HS  RIGHT |  | LACUNAR INFACTS  MILD AD | | AMYGDALA | MI |
|  | EPM14 |  | 47 | F | TYPE 1 HS  LEFT |  | NONE | | AMYGDALA | UNKNOWN HEAD ONLY |
|  | EPM15 |  | 18 | M | NO HS |  | PATH NEG | | AMYGDALA | SUDEP |
|  | EPM16 |  | 67 | F | TYPE 1 HS  LEFT |  | CORTICAL SCAR  CEREBELLAR ATROPHY | | AMYGDALA | SUDEP |
| POST MORTEM EPILEPSY CONTROLS | C1 | NA | 58 | F | NO HS | - | PATH NEG | | HIPPOCAMPAL BODY | IHD |
|  | C2 | NA | 57 | F | NO HS | - | PATH NEG | | HIPPOCAMPAL BODY | PANCREATITIS |
|  | C3 | NA | 75 | M | NO HS | - | PATH NEG | | HIPPOCAMPUS BODY (BOTH SIDES) | UK |
|  | C4 | NA | 28 | M | NO HS | - | PATH NEG | | HIPPOCAMPUS BODY (BOTH SIDES),TEMPORAL CORTEX, AMYGDALA | CARDIAC DEATH |
|  | C5 | NA | 36 | M | NO HS | - | PATH NEG | | HIPPOCAMPUS BODY | UK |
|  | C6 | NA | 56 | M | NO HS | - | PATH NEG | | HIPPOCAMPUS BODY, AMYGDALA , TEMPORAL LOBE | IHD |
|  | C7 | NA | 57 | M | NO HS | - | PATH NEG | | HIPPOCAMPUS BODY, AMYGDALA , TEMPORAL LOBE | IHD |
|  | C8 | NA | 49 | M | NO HS | - | PATH NEG | | HIPPOCAMPUS BODY, AMYGDALA , TEMPORAL LOBE | IHD |
|  | C9 | NA | 62 | F | NO HS | - | PATH NEG | | HIPPOCAMPUS BODY, AMYGDALA | ADENOID CYSTIC CARCINOMA |
|  | C10 | NA | 85 | F | NO HS | - | PATH NEG | | AMYGDALA |  |
|  | C11 | NA | 64 |  | NO HS | - | PATH NEG | | TEMPORAL LOE AND HIPPOCAMPUS |  |
|  | C12 | NA | 50 | M | NO HS | - | PATH NEG | | AMYGDALA | IHD |

**Supplemental Table 1. Detail of each case used in the study including clinical and psychometric data and the type of study carried out.** The cases are numbered with the following nomenclature : EA = epilepsy adult surgical, EP=epilepsy paediatric surgical, EPM=epilepsy post-mortem tissue, C = control post-mortem tissue. Seizure types have been recorded using the nomenclature on their records. Aura + = documentation of an aura preceding seizure of any type. Aura- no aura documented. PS= partial seizures, CPS= complex partial seizures, GS= generalised seizures including secondary generalised seizures, GTCS = generalised tonic-clonic seizures, AM = automotor seizures, AbS = absence seizures, MJ=myoclonic jerks, FS = febrile seizures. TLE = temporal lobe epilepsy, RE= refractory epilepsy no further detail of seizures, SFE= syndrome of symptomatic focal epilepsy, U/C = epilepsy syndrome unclassified, HS= hippocampal sclerosis, GNT = graded naming tests, IF=immunofluorescence, PM = post mortem, LEAT = long-term epilepsy-associated tumour, IHD = ischaemic heart disease, UK= unknown, CVD = cerebrovascular disease, PE = pulmonary embolus. ~Details of psychometric tests and grading of moderate or severe deficit in supplementary methods. IHCq= quantitative immunohistochemistry, IF=Immunofluorescence
