# Supplementary material for: Doublecortin-expressing cell types in temporal lobe epilepsy
Source: Acta Neuropathol Commun. 2018 Jul 13;6:60. doi: 10.1186/s40478-018-0566-5 (PMC6045867; doi:10.1186/s40478-018-0566-5)
Supplement: Supplementary file 2 — Supplementary Methods (DOCX 18 kb) [file 40478_2018_566_MOESM2_ESM.docx]

Supplemental file

Methods

**Immunohistochemistry**

Immunofluorescence double labeling : Sections were incubated overnight at 4 ºC, and on the next day, species-specific HRP secondary solution (Vector Laboratories Inc., Peterborough, UK) was applied for 30 minutes, before fluorescein-labelled antibody in tyramide signal amplification (TSA) buffer (1:500, Perkin Elmer, Massachusetts, UK) was applied for eight minutes. Sections were thoroughly washed using phosphate buffer saline (PBS), and then immersed in 0.9% hydrogen peroxide solution and 10 % normal horse serum in PBS for ten minutes. On the following day, species-specific HRP secondary solution (Vector Laboratories Inc., Peterborough, UK) was applied for 30 minutes, before rhodamine-conjugated antibody in TSA buffer was applied for eight minutes. After PBS washes, sections were cover-slipped using mounting medium (Vector Laboratories Inc., Peterborough, UK). The protocol differed for anti-GFAP delta, where this antibody was incubated on sections for 48 hours at 4ºC, and for anti-Iba1, anti-Sox2, anti-CD34 and anti-GFAP, where goat anti-rabbit alexa-546 conjugated secondary antibodies (1:200, Life Technology, Paisley, UK) was applied for one hour at room temperature. Details of dilutions and antibody sources and pre-treatments are detailed in Table 2.

**Psychometry**

Pre-operative memory performance was assessed as previously described ([Thompson *et al.*, 2015](#_ENREF_15)). Memory tests for verbal and visual memory included the List Learning and Design Learning subtests from the Adult Memory & Information Processing Battery (AMIPB) prior to 2007 and the BIRT Memory and information processing battery (BIMPB) from 2007 onwards, as part of the standard psychometric assessments for all patients being considered for epilepsy surgery. The psychometric measurements included verbal and visual task memory score tests ([Baxendale *et al.*, 2006](#_ENREF_1)) in addition to the graded naming test (GNT) ([Bird & Cipolotti, 2007](#_ENREF_2)), to assess naming capacity, which has been found to be sensitive to dominant temporal lobe resections ([Bonelli *et al.*, 2012](#_ENREF_6)). A patient was classified as having a deficit pre-operatively if scoring they scored 1 SD or more below average, and as having a severe deficit if they scored 2 SD or more below average (Supplemental Table 1 for results).
