# Supplementary figures and images for: Doublecortin-expressing cell types in temporal lobe epilepsy
Source: Acta Neuropathol Commun. 2018 Jul 13;6:60. doi: 10.1186/s40478-018-0566-5 (PMC6045867; doi:10.1186/s40478-018-0566-5)

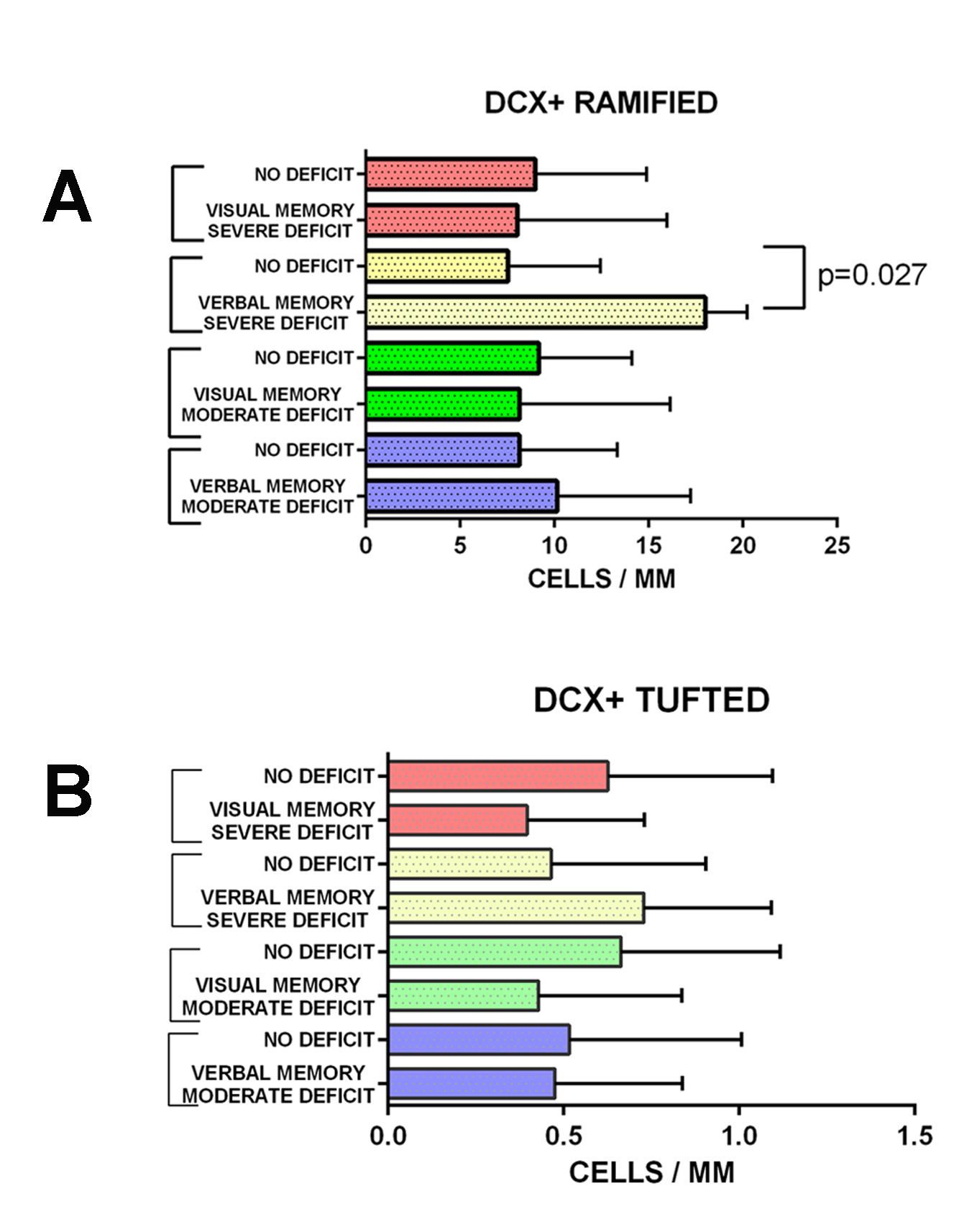

Supplement: Supplementary file 3 — Figure S1. Bar graphs of the relationship between pre-operative memory function and DCX+ cells in the superficial temporal lobe. A. There was an association between severe memory deficit and increased ramified type DCX+ cells (p = 0.027). However the number of patients in this series is small and requires validation with a larger cohort. B. There was no association between the number of tufted DCX+ neuronal cells in layer II of the temporal cortex and pre-operative memory function. (JPG 181 kb) [file 40478_2018_566_MOESM3_ESM.jpg]

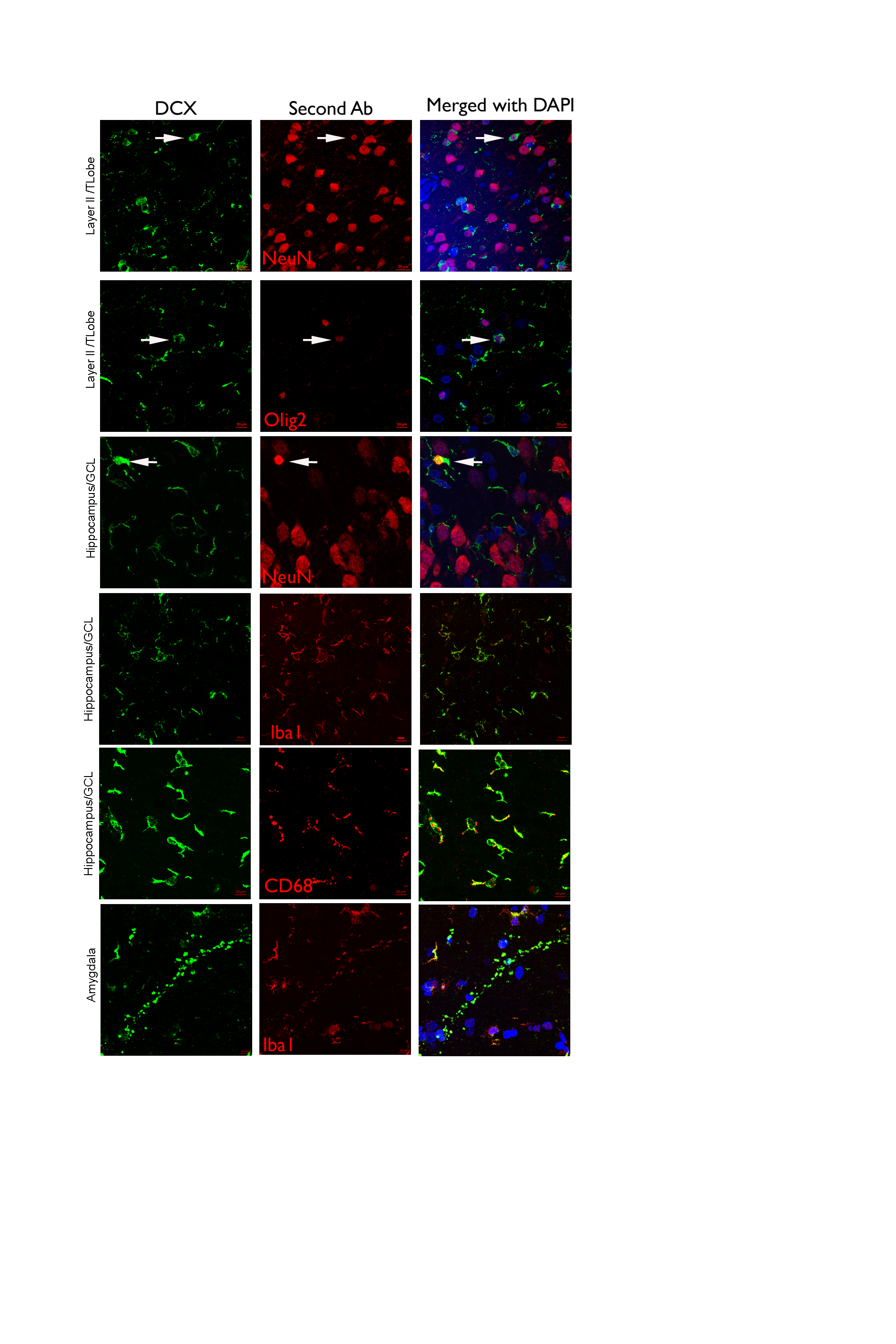

Supplement: Supplementary file 4 — Figure S2. Split channels for DCX double labelling as indicated in temporal lobe, hippocampus and amygdala for images shown in Fig. 3. (TIF 15727 kb) [file 40478_2018_566_MOESM4_ESM.tif]
